# Why RGB Imaging Should be Used to Analyze *Fusarium Graminearum* Growth and Estimate Deoxynivalenol Contamination

**DOI:** 10.3390/mps2010025

**Published:** 2019-03-18

**Authors:** Edgar Cambaza, Shigenobu Koseki, Shuso Kawamura

**Affiliations:** 1 Laboratory of Food Process Engineering, Graduate School of Agriculture, Hokkaido University, Sapporo 060-0808, Japan; koseki@bpe.agr.hokudai.ac.jp (S.K.); shuso@bpe.agr.hokudai.ac.jp (S.K.); 2 Department of Biological Sciences, Faculty of Sciences, Eduardo Mondlane University, Av. Julius Nyerere, nr. Maputo 3453, Mozambique

**Keywords:** RGB, *Fusarium graminearum*, growth, deoxynivalenol

## Abstract

Size-based fungal growth studies are limited because they do not provide information about the mold’s state of maturity, and measurements such as radius and diameter are not practical if the fungus grows irregularly. Furthermore, the current methods used to detect diseases such as *Fusarium* head blight (FHB) or mycotoxin contamination are labor-intensive and time consuming. FHB is frequently detected through visual examination and the results can be subjective, depending on the skills and experience of the analyzer. For toxin determination (e.g., deoxynivalenol (DON), the best methods are expensive, not practical for routine. RGB (red, green and blue) imaging analysis is a viable alternative that is inexpensive, easy to use and seemingly better if enhanced with statistical methods. This short communication explains why RGB imaging analysis should be used instead of size-based variables as a tool to measure growth of *Fusarium graminearum* and DON concentration.

## 1. Introduction

The last two decades have witnessed a rapid global re-emergence of *Fusarium*, an important genus of cereal pathogens [1,2,3], due to climate change [4], edaphic and agro-technical factors [5,6,7]. These molds have garnered attention from researchers, scholars and legislators because of their deleterious impact in agriculture, trade, health and animal sciences. The most commonly found in temperate areas are *F. graminearum* and *F. moniliforme*, sometimes *F. culmorum*, *F. proliferatum*, *F. equiseti*, *F. sporotrichoids* and *F. poae* [8,9,10]. *F. graminearum* is perhaps the main mold responsible for *Fusarium* head blight (FHB) in crops such as oats (*Avena sativa*), rice (*Oryza sativa*) and other cereals [5]. This disease is responsible for yield losses, reduced nutritive, physical and chemical quality of seed and the consequent difficulties for the marketing, exporting and processing of infected grains [1,10,11,12]. Furthermore, *F. graminearum* produces several mycotoxins from which the most frequently detected is deoxynivalenol (DON) [13,14].

DON (also called vomitoxin or 3,7,15-trihydroxy-12,13-epoxytrichothec-9-en-8-one) is a trichothecene, a group of toxins recognized among the most powerful inhibitors of protein and DNA synthesis [3,15,16,17]. Vomitoxin was first found in Japanese barley, then characterized by Morooka, et al. [18]. Its consumption was found to result in animal disease, but there are also numerous records of human intoxication [19]. In pigs from all age groups and other non-ruminants [14], DON is well known for causing nausea, vomiting, diarrhea, immunosuppression, toxicity to the nervous system, embryo and teratogenic effects, feed refusal, reduction in weight and sometimes death [3,10,14,15,17,20,21,22]. There are records of human intoxication in India, China and at least eight outbreaks in Japan during the 20th century [15,23], with people affected manifesting gastrointestinal upset, dizziness, vomiting and headaches, among other symptoms [21]. For this reason, it is necessary to prevent DON from entering the food chain, and a key factor in this is knowing how to control *F. graminearum* [1,24].

**Figure 1:**
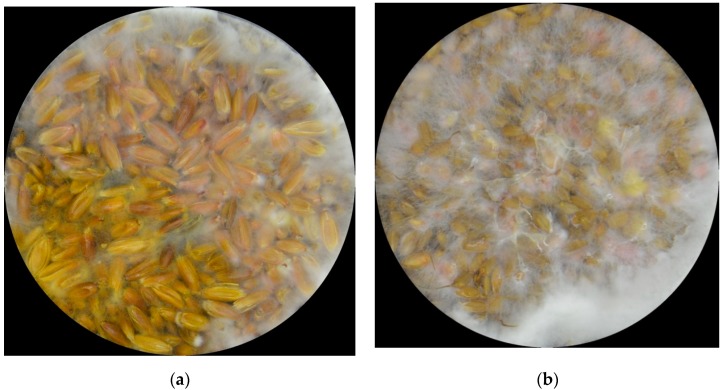
*Fusarium graminearum* at a_w_ = 0.99 after 8 days in heavily contaminated (**a**) oats and (**b**) rice.

When *F. graminearum* affects grains, it shows fairly well-known symptoms (Figure 1). The grains become smaller, shriveled, and the surface becomes pale “like covered with white chalk”, frequently showing pink coloration [6,13,25]. These characteristics are often screened by trained personnel. According to Goswami and Kistler [11], the fungus overwinters as a saprobe (white mycelium) in dead leaves, growing in decaying leaves where it starts producing conidia (orange and pink) and perithecia (purple) just before it invades a living host.

All of these structures present specific colors, and this knowledge could be used for researchers’ and farmers’ advantages as a tool to analyze the quality of grains, and to possibly estimate the quantity released by the parasite in food or feed through digital imaging. Indeed, Dammer, et al. [19] detected FHB infected grains using RGB imaging analyses combined with statistical approaches, and Jirsa and Polišenská [25] went one step further and distinguished samples containing DON, though they just compared samples heavily damaged by FHB with healthy ones without considering any intermediate stages or how environmental or nutritional factors might be involved.

This paper explains why colors developed by *F. graminearum* throughout its lifecycle can be used as a tool to analyze its growth pattern, as well as to estimate the quantity of DON it releases to the food matrix. There are some limitations of size-based models, particularly because (1) they do not provide information about the metabolic condition of the mold, especially during stages at which there is no expansion in size (such as lag and stationary stages) and (2) the most common size-based variables (radius, diameter) cannot accurately represent growth when the mold presents irregular forms [26].

## 2. Limitations of the Current Models

Since there are two issues to consider (FHB and DON), it is important to know which are the problems regarding them and why the status quo still needs to improve. Regarding FHB, in several parts of the world infected kernels are still separated from damaged kernels through visual evaluation by buyers and trained inspectors [13], a process that is fast but labor intensive, subjective and offers low repeatability, as well as being ultimately inconsistent and not suitable for a large amount of samples [1,13,25]. In the case of DON, the standard analytical methods are destructive, expensive, time consuming and not appropriate for screening [1,13,27]. For these reasons, there is a need for simple, low-priced and reliable ways to assess the quality of grains, predict and prevent FHB, if possible, the presence of DON. A very good approach would be the development of models based on the variables more likely to be associated with *F. graminearum* infection and DON contamination [19].

**Figure 2:**
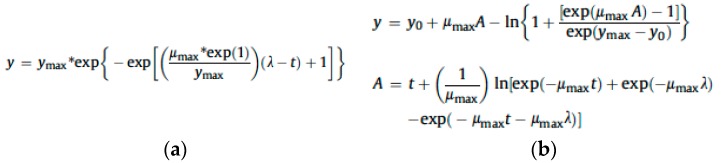
(**a**) Gompertz and (**b**) Baranyi growth models. Notes: y = size; y_max_ = maximum size; y_0_ = initial size; λ = lag time; μ_max_ = maximum growth rate; t = time.

In 2009, Garcia, et al. [28] wrote a very elucidating review on the major analytical approaches used the predict the level of mycotoxins in food. The first part of the article focuses on predictive models for mold germination growth and inactivation. According to the authors, the major growth methods used for molds are derived from bacterial studies: lineal, Gompertz and Baranyi (Figure 2). Though these models are easy to use and practical in some situations, they require spatial variables such as radius or diameter. However, fungal growth is more complex than mere spatial expansion.

For instance, when *F. graminearum* grows on solid agar in a Petri dish and reaches its full size, it can no longer expand, but the fungus is still alive, with the ability to grow further, produce spores and propagate. In these cases, the size has no more use. Other impractical aspects are cases in which the mold grows in an irregular fashion. Garcia, et al. [28] also mentioned the quantification of ergosterol in a medium to estimate fungal growth. Though the idea is good and certainly provides information on the mold’s metabolism, it would be preferable to use methods less destructive or just less invasive.

Regarding models for toxin quantification, they were also described, but the problem persists. Most models use variables such as time, temperature and a_w_ to predict toxin concentration, but they have the same sort of limitation as the size-based approach: they lack information about the mold’s metabolic state. Time and environmental variables are not inherent characteristics of an organism, although they usually play a significant role in its behavior. In order to effectively affect the organism, they have to go through a barrier of homeostatic forces. Growth indicators coming from within the organism are perhaps a best approach to really understand them.

Fungal pigmentation makes more sense than most approaches mentioned by Garcia, et al. [28] as a variable to analyze its state of development and the organism’s metabolism [26]. Pigmentation does not necessarily need to be directly related to toxin production. It just needs variations with significant statistical or mathematical correlations. Indeed, there is a body of evidence demonstrating the feasibility of image-based approaches to obtain reliable information about the growth of *F. graminearum* and DON synthesis, some of which Saccon, et al. [1] have described in detail.

## 3. RGB Imaging in Plant Pathology

Optical techniques are among the best way to assess large numbers of samples in a fast and non-destructive way [1]. According to Gupta, et al. [29], the main types of imaging techniques are photometric feature-based (also called RGB), fluorescence, hyperspectral and thermal. Padmavathi and Thangadurai [30] describe an RGB image as representing three color component intensities (red, green and blue), and RGB-based methods have been broadening their applications in several areas of agronomic sciences because of their fitness to analyze color discrepancies between distinct biological samples [13,29,30].

RGB imaging is progressively replacing human vision in the evaluation of food quality [29] because it provides the investigator with new tools of higher quality to reliably analyze the commodity’s shape, intensity of colors of several plant structures including seeds [13]. Furthermore, these methods are relatively easy and inexpensive [19,31], particularly when used with statistics [32]. According to Wiwart, et al. [13], this technique can be effectively used to effectively evaluate grains and assess DON content through the frequency of *Fusarium* damaged kernels (although in their study they detected RGB values and then converted them to HSI (hue, saturation, intensity), perhaps because they were more concerned with those variables).

**Figure 3:**
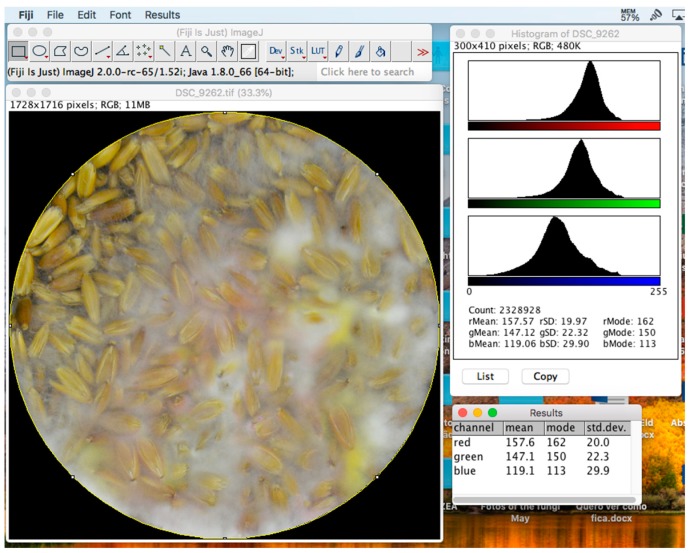
ImageJ toolbox (top left), a photo of heavily contaminated oat grains (bottom left), a color histogram showing distributions of intensities of RGB components in the photo (top right), and the average intensity of each color (bottom right).

ImageJ (National Institutes of Health, Bethesda, Maryland, US; Figure 3) is an open-source Java-based imaging program [33] worth mentioning, as it has been contributing widely to imaging analysis for over 25 years [34]. It had originally been developed for medical research, but its tools can be applied in virtually any field requiring imaging analysis. The software has a very intuitive interface with very effective embedded plugins and a website from which it is possible to download more, mostly created by independent developers. One of the plugins, Color Histogram [35], is suitable for imaging analysis in agriculture and it can be used to determine average RGB values of grains, fungi or any other sort of image.

## 4. RGB Imaging and *F. graminearum*

This method was tested in a four-year study in the Laboratory of Agricultural and Food Process Engineering, Graduate School of Agriculture, Hokkaido University, Japan.

### 4.1. How were the Experiments Performed?

#### 4.1.1. Mold Isolate

This study used an *F. graminearum* isolate from the catalogue of the Japan Collection of Microorganisms (JCM). It was registered as the teleomorph *Gibberella zeae* (Schwabe) Petch, strain TH-5, isolated by Sugiura [36] from rice stubble in Hirosaki, Aomori Prefecture, Japan. It is a known producer of deoxynivalenol, 15-acetyldeoxinivalenol, and zearalenone [37]. The inocula were produced by growing a specimen of yeast extract agar (YEA) in a Petri dish. Each inoculum consisted of a small cube of approximately 1 cm^3^ of agar containing the fungus, taken from the medium.

#### 4.1.2. Incubation and RGB Determination

The cereals were split into three groups, each with distinct water activity (a_w_). For oats and YEA, groups had a_w_ = 0.94, 0.97 and 0.99; for rice, 0.97, 0.98 and 0.99. In total, each cereal was used for the preparation of 153 media in Petri dishes, 144 to grow the molds and 9 as control to determine the colors in the beginning on the experiment. Thus, 288 specimens of *F. graminearum* were sown, one in each medium. In the end, there were three replicates per water activity in which five repetitions were incubated at four different temperatures: 15, 20, 25 and 30 °C. From the fourth day of incubation, three replicates per temperature, cereal and a_w_ were taken for DON quantification. Before the extraction, the fungi were photographed vertically in a black bucket from 30 cm above. The camera model was Nikon D3200 with a DX SWM VR lens (Nikon Corporation, Tokyo, Japan), and it was used without a flash or any automation affecting illumination. The only source of light was a round LED attached to the bucket’s lid. The photos were then processed using the method described by Cambaza, et al. [26] on ImageJ software (FIJI edition, National Institutes of Health, Bethesda, MD, USA), developed by the National Institutes of Health and the Laboratory for Optical and Computational Instrumentation (LOCI, University of Wisconsin) [34]. ImageJ allowed the determination of average intensities of the RGB components from the photos.

The analysis considered only the fungal surface, excluding any background including the plate borders or agar. In the end, the variables to analyze were the incubation time (in days), a_w_ and the RGB parameters, converted from the eight-bit notation (0−255) to the arithmetic index (0.0−1.0).

#### 4.1.3. Extraction and High-Performance Liquid Chromatography (HPLC)

For extraction, each sample was mixed with 100 mL of water:acetonitrile (84:16) and blended in a Seward Stomacher 400 machine (Seward Ltd., Singapore). Approximately 15 mL of the filtered extract was transferred to a tube and 2 mL of this filtrate were purified using Supel TOX DON cartridges [19]. These cartridges eliminate undesirable fat, pigment and carbohydrates and retain large molecules. HPLC was run through a Jasco CrestPak C18T-5 affinity column using (a) water:acetonitrile:methanol (92:4:4) and (b) acetonitrile as a mobile phase with a flow rate of 0.2 mL/min at 35 °C and detection set for ultraviolet light at 220 nm. DON peaks consistently appeared at 8 min.

#### 4.1.4. Statistical Analysis

The statistical analysis was performed on JASP 0.9 (The JASP Team, Amsterdam, Netherlands), Jamovi 0.9 (Jamovi Project, Amsterdam, Netherlands) and Microsoft Excel (Version 14.5.8, Microsoft, Redmond, WA, USA). All the hypotheses tested were carried out with α = 0.05. The distribution of intensities of red, green and blue and DON concentration were compared through analysis of covariance (ANCOVA) to find if their differences were significant, and if necessary followed by Tukey’s post-hoc comparisons. Finally, the correlations between the colors and DON concentration were analyzed through scatter plot matrices.

### 4.2. Summary of Major Findings

**Figure 4:**
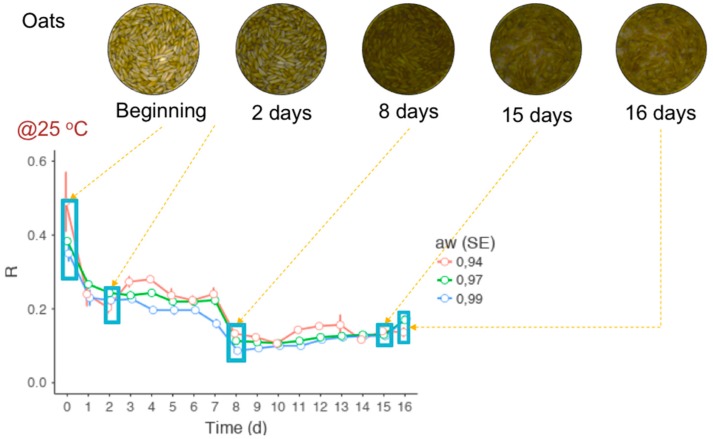
Red color variation of *F. graminearum*-contaminated oats over 16 days. Note that the color variation was similar, regardless of a_w_.

Considerable parts of the experiment have been published in journal articles [8,26,38,39,40] and conferences [41,42]. There are two unpublished works still under peer review, one of them with a preprint [43]. After a careful examination of pros and cons regarding the use of color alternatives to size-based growth measurements of *F. graminearum*, as well as also to predict how much DON the mold produces at a particular time, RGB imaging seems to bear potential to perform the task in several circumstances. Colors of *F. graminearum* are very predictable (Figure 4) because they derivate from considerably few sources, and among them aurofusarin and white mycelium seem to be the most influential. Their properties, and how the most important environmental factors affect them, seem to be known well enough to allow the development of reliable RGB imaging approaches. Favorable temperature, a_w_ and availability of nutrients stimulate high-density biomass and production of pigmented spores and perithecia. It was possible to design a RGB imaging-based growth model with a mycelial and a sporular stage, both initiated by lag stages. The rapidity at which each occurs appears to be a function of the type of nutrient. The intensity is likely to respond to how optimal or harsh the temperature and a_w_ for growth are.

### 4.3. Recommendations

RGB-based methods have high potential beyond the scope of this particular study. Future researchers should consider the expansion of this idea for other strains of *F. graminearum* or even other species or genera, analyzing other toxins, metabolites, plant diseases, growth media, temperature and a_w_ ranges. Color-based models can be further explored and possibly incorporated with sorters to separate grains with a high likeliness of having mycotoxins.

Other technical features of the experiment can be improved. For instance, a very good light-isolating chamber could replace the bucket used to better control possible interference. Even the illumination itself could be optimized. The experiment could be reproduced using simpler everyday cameras, such as smartphones, so that a farmer or common citizen could have the ability to monitor *Fusarium* infection or DON contamination at home.

Good agricultural practices are very important. *F. graminearum* is highly resistant, and sometimes grains can look safe but the mold is there. For instance, there were cases where grains with low water activity did not show any growth until the eighth day of incubation and were contaminated. Yet, it is better to maintain the grain at suboptimal conditions to avoid mold. If the grains are heavily infected or exhibit pink or yellow colors on the surface, it is better to avoid them.

### 4.4. Benefits of this Approach

Cereals are arguably the most important food. Rice alone is “the predominant staple food for 17 countries in Asia and the Pacific, nine countries in North and South America, and eight countries in Africa”, according to the Food and Agriculture Organization of the United Nations (FAO) [21], and Olivares Diaz [44] described this commodity as the major caloric source for half of the human race. A cereal pathogen with the potential to destroy high-yielding crops in a few weeks of harvest reduce considerably the quality of kernels and spread toxins through the grains [11] is a major threat to food security, safety and quality. This is the reason why the European Union issued maximal residual limits (1.25 μg/g) for DON in 2006, and Japan set provisional limits (1.1 μg/g).

Research on imaging analysis to better understand FHB and DON has already undergone remarkable progress, but there is still plenty of work to improve its methods. Besides the insightful review by Saccon, et al. [1] and the paper on RGB analysis by Jirsa and Polišenská [25] and Dammer, et al. [19], there are other works more or less directly related to this thesis’ topic. For instance, Ruan, et al. [45] combined RGB imaging with a neural network to discriminate infected kernels, and Wiwart, et al. [13] used Digital image analysis to detect FHB in triticale. Yet, none of these authors have so far analyzed the entire process of infection in a gradual or longitudinal fashion, including the pattern of color variation as the mold expands as mycelium, matures and produces different types of spores. No one has yet tried to confront in a meticulous way the colors side by side with DON contamination. It is necessary to perform a meticulous analysis across nutrient sources, temperatures and a_w_.

RGB imaging analysis provides a novel perspective in which the color of *F. graminearum* is treated the same way as the main stream researchers use mycelial radius or diameter as a growth measure. No approach is right or wrong, but each has its advantages and disadvantages. It is reasonable or even intuitive to regard spatial variables as growth measures because humanity has been using height, length, thickness, perimeter, diameter, etc. to evaluate the growth of a plant, mammal, fish or any imaginable organism. It might be a good time to explore ideas “outside the box”. RGB-based analysis will subsidize the comprehension of the biology and ecology for *F. graminearum* and plant pathogens in general, and is expected to be useful for scholars, researchers, farmers, the industry, policymakers and society.

## 5. Final Remarks

The development of a viable imaging tool to analyze the growth pattern of *F. graminearum* and production of DON is necessary, as metabolic activity be fairly understood through basic principles underlying the mold’s ecology, optics and statistics. This seems very feasible, but also requires very careful scrutiny. From recent studies [26,38,39,41], some aspects were observed: colors of *F. graminearum* result from a combination of unpigmented biomass and a considerably small variety of pigments, all produced according to the organism’s adaptive strategies; there is a predictable pattern of color variation throughout the mold’s lifecycle, easy to analyze through RGB imaging; *F. graminearum* is highly sensitive to temperature, a_w_, nutrients and several other environmental factors; the growth pattern of this mold is basically the same across different types of substrates, but the easiness to obtain nutrients affects the mold’s growth strategy and the speed at which the organism thickens the mycelium or produces spores.

*F. graminearum* quickly adapts to different environments. Some authorities [5,11] have provided valuable accounts on the mold’s lifecycle and facilitated the comprehension of the phenomena observed in the current experiment. The fungus has two basic growth strategies, and these can be related to *r* and *k* survival strategies as described by Taylor, et al. [46]: As the organism reaches a substrate, it quickly produces a mycelium and spreads through the medium (*k*-strategy). If the medium is rich in nutrients, the fungus favors the production of spores (*r*-strategy). *K*-strategy results in an increase of levels of all RGB components as the mold becomes white. *R*-strategy increases the RGB components if the medium is dark, acidic (under low pH the mold produces yellow pigments) or highly illuminated (light stimulates the production of carotenoids), or decreases RGB if the medium is bright, for instance if there is high mycelial biomass. Toxin production occurs mainly when the fungus adopts the *r*-strategy (i.e., when there are live colors) [11] and this is indeed one of the ways to detect *Fusarium* head blight. Since the growth stages or strategies are always associated with particular colors, they are predictable.

It is also important to consider the role of temperature and a_w_ in the dynamics of RGB changes during the growth of *F. graminearum* [47]. There are two major profiles, determined by harsh or optimal environmental factors, with intermediate states. When the conditions tend to be optimal (25 °C and a_w_ = 0.99), the fungus favors rapid growth and production of spores (*r*-strategy). Thus, one can expect a very rapid rise in all RGB values (either from pigmentation or high mycelial biomass) and also DON concentration. RGB imaging is highly reliable in these situations, especially if there are no strong natural barriers for colonization, such as husk. This explains why there were high correlations between colors and DON concentration in rice samples containing peeled grains and agar, but not as much in oats. Under severe conditions, the fungus colonizes as fast but the mycelium does not thicken much (possibly to save energy), and it might even be hardly noticeable in grains. In these cases, imaging analysis might not help much, but the chances for the grains to be contaminated are also scarcer. Thus, RGB analysis seems to be low cost with a rapid approach when conditions seem suspicious.

## References

[1] Saccon F.A., Parcey D., Paliwal J., Sherif S.S. (2017). Assessment of *Fusarium* and deoxynivalenol using optical methods. Food Bioprocess Technol..

[2] Velluti A., Sanchis V., Ramos A.J., Turon C., Marin S. (2004). Impact of essential oils on growth rate, zearalenone and deoxynivalenol production by *Fusarium graminearum* under different temperature and water activity conditions in maize grain. J. Appl. Microbiol..

[3] Ryu D., Bullerman L.B. (1999). Effect of cycling temperatures on the production of deoxynivalenol and zearalenone by *Fusarium graminearum* NRRL 5883. J. Food Prot..

[4] Fredlund E., Gidlund A., Sulyok M., Borjesson T., Krska R., Olsen M., Lindblad M. (2013). Deoxynivalenol and other selected *Fusarium* toxins in Swedish oats—Occurrence and correlation to specific *Fusarium* species. Int. J. Food Microbiol..

[5] Leplat J., Friberg H., Abid M., Steinberg C. (2013). Survival of *Fusarium graminearum*, the causal agent of *Fusarium* head blight. A review. Agron. Sustain. Dev..

[6] Jaillais B., Roumet P., Pinson-Gadais L., Bertrand D. (2015). Detection of *Fusarium* head blight contamination in wheat kernels by multivariate imaging. Food Control.

[7] Langseth W., Elen O. (1996). Differences between barley, oats and wheat in the occurrence of deoxynivalenol and other trichothecenes in Norwegian grain. J. Phytopathol..

[8] Cambaza E.M., Koseki S., Kawamura S. (2018). Meta-analytic review on the impact of temperature and water activity in deoxynivalenol synthesis by *Fusarium graminearum*. Food Res..

[9] Dvořáček V., Prohasková A., Chrpová J., Štočková L. (2012). Near infrared spectroscopy for deoxynivalenol content estimation in intact wheat grain. Plant Soil Environ..

[10] Sabater-Vilar M., Malekinejad H., Selman M.H., van der Doelen M.A., Fink-Gremmels J. (2007). In vitro assessment of adsorbents aiming to prevent deoxynivalenol and zearalenone mycotoxicoses. Mycopathologia.

[11] Goswami R.S., Kistler H.C. (2004). Heading for disaster: *Fusarium graminearum* on cereal crops. Mol. Plant Pathol..

[12] Shahin M., Hatcher D., Symons S. (2012). Development of multispectral imaging systems for quality evaluation of cereal grains and grain products. Computer Vision Technology in the Food and Beverage Industries.

[13] Wiwart M., Koczowska I., Borusiewicz A. (2011). Estimation of *Fusarium* head blight of triticale using digital image analysis of grain. Proceedings of International Conference on Computer Analysis of Images and Patterns.

[14] Cavret S., Laurent N., Videmann B., Mazallon M., Lecoeur S. (2010). Assessment of deoxynivalenol (DON) adsorbents and characterisation of their efficacy using complementary in vitro tests. Food Addit. Contam..

[15] Martins M.L., Martins H.M. (2002). Influence of water activity, temperature and incubation time on the simultaneous production of deoxynivalenol and zearalenone in corn (*Zea mays*) by *Fusarium graminearum*. Food Chem..

[16] Greenhalgh R., Neish G.A., Miller J.D. (1983). Deoxynivalenol, acetyl deoxynivalenol, and zearalenone formation by Canadian isolates of *Fusarium graminearum* on solid substrates. Appl. Environ. Microbiol..

[17] He J., Yang R., Zhou T., Tsao R., Young J.C., Zhu H., Li X.Z., Boland G.J. (2007). Purification of deoxynivalenol from Fusarium graminearum rice culture and mouldy corn by high-speed counter-current chromatography. J. Chromatogr. A.

[18] Morooka N., Uratsuji N., Yoshisawa T., Yamamoto H. (1972). Studies on the toxic substances in barley infected with *Fusarium* spp.. Food Hyg. Saf. Sci..

[19] Dammer K.-H., Möller B., Rodemann B., Heppner D. (2011). Detection of head blight (*Fusarium* ssp.) in winter wheat by color and multispectral image analyses. Crop Prot..

[20] Garcia D., Barros G., Chulze S., Ramos A.J., Sanchis V., Marin S. (2012). Impact of cycling temperatures on Fusarium verticillioides and Fusarium graminearum growth and mycotoxins production in soybean. J. Sci. Food Agric..

[21] Almeida M.I., Almeida N.G., Carvalho K.L., Goncalves G.A., Silva C.N., Santos E.A., Garcia J.C., Vargas E.A. (2012). Co-occurrence of aflatoxins B(1), B(2), G(1) and G(2), ochratoxin A, zearalenone, deoxynivalenol, and citreoviridin in rice in Brazil. Food Addit. Contam..

[22] Bergsjo B., Langseth W., Nafstad I., Jansen J.H., Larsen H.J. (1993). The effects of naturally deoxynivalenol-contaminated oats on the clinical condition, blood parameters, performance and carcass composition of growing pigs. Vet. Res. Commun..

[23] Yoshizawa T. (2013). Thirty-five Years of Research on Deoxynivalenol, a Trichothecene Mycotoxin: With Special Reference to Its Discovery and Co-occurrence with Nivalenol in Japan. Food Saf..

[24] Langseth W., Stabbetorp H. (1996). The effect of lodging and time of harvest on deoxynivalenol contamination in barley and oats. J. Phytopathol..

[25] Jirsa O., Polišenská I. (2014). Identification of *Fusarium* damaged wheat kernels using image analysis. Acta Univ. Agric. Silvic. Mendel. Brun..

[26] Cambaza E., Koseki S., Kawamura S. (2018). The Use of Colors as an Alternative to Size in *Fusarium graminearum* Growth Studies. Foods.

[27] Benaliaa S., Bernardi B., Cuberob S., Leuzzic A., Larizzac M., Blascob J. (2015). Preliminary trials on hyperspectral imaging implementation to detect mycotoxins in dried figs. Chem. Eng..

[28] Garcia D., Ramos A.J., Sanchis V., Marin S. (2009). Predicting mycotoxins in foods: A review. Food Microbiol..

[29] Gupta S.D., Ibaraki Y., Trivedi P. (2014). Applications of RGB color imaging in plants. Plant Image Anal. Fundam. Appl..

[30] Padmavathi K., Thangadurai K. (2016). Implementation of RGB and grayscale images in plant leaves disease detection–comparative study. Indian J. Sci. Technol..

[31] Teena M., Manickavasagan A., Al-Sadi A.M., Al-Yahyai R., Deadman M. (2016). RGB color imaging to detect *Aspergillus flavus* infection in dates. Emir. J. Food Agric..

[32] Yoon S.-C., Shin T.-S., Heitschmidt G.W., Lawrence K.C., Park B., Gamble G. Hyperspectral imaging using a color camera and its application for pathogen detection. Proceedings of the Image Processing: Machine Vision Applications VIII.

[33] Abràmoff M.D., Magalhães P.J., Ram S.J. (2004). Image processing with ImageJ. Biophotonics Int..

[34] Schneider C.A., Rasband W.S., Eliceiri K.W. (2012). NIH Image to ImageJ: 25 years of image analysis. Nat. Methods.

[35] Burger W., Burge M.J. (2016). Digital Image Processing: An Algorithmic Introduction Using Java.

[36] Sugiura Y., Japan Collection of Microorganisms (1996). *Gibberella zeae* (Schwabe) Petch. JCM Catalogue.

[37] Sugiura Y., Watanabe Y., Tanaka T., Yamamoto S., Ueno Y. (1990). Occurrence of *Gibberella zeae* strains that produce both nivalenol and deoxynivalenol. Appl. Environ. Microbiol..

[38] Cambaza E. (2018). Comprehensive Description of *Fusarium graminearum* Pigments and Related Compounds. Foods.

[39] Cambaza E., Koseki S., Kawamura S. (2018). *Fusarium graminearum* Colors and Deoxynivalenol Synthesis at Different Water Activity. Foods.

[40] Cambaza E., Koseki S., Kawamura S. (2019). *Fusarium graminearum* growth and its fitness to the commonly used models. Int. J. Agric. Environ. Food Sci..

[41] Cambaza E., Koseki S., Kawamura S. *Fusarium graminearum* colors and deoxynivalenol synthesis at different temperatures. Proceedings of the 2nd International Conference on Food Quality, Safety and Security (FOOD QualSS).

[42] Cambaza E., Koseki S., Kawamura S. Comprehensive description of Fusarium graminearum pigments. Proceedings of the 8th Academic Exchange for Collaborative Research Between ETHZ and Hokkaido University (AECoR8).

[43] Cambaza E., Shigenobu K., Kawamura S. (2019). RGB Imaging as Tool to Analyze the Growth of Fusarium graminearum in Infected Oats (*Avena sativa*) and Rice (*Oryza sativa*). Preprints.

[44] Olivares Diaz E. (2018). Physical and Chemical Properties of Multiple Varieties of NERICA, Indica and Japonica Types of Rice for Assessing and Enhancing Quality.

[45] Ruan R., Ning S., Song A., Ning A., Jones R., Chen P. (1998). Estimation of *Fusarium* scab in wheat using machine vision and a neural network. Cereal Chem..

[46] Taylor D.R., Aarssen L.W., Loehle C. (1990). On the relationship between r/K selection and environmental carrying capacity: A new habitat templet for plant life history strategies. Oikos.

[47] Choi S., Jun H., Bang J., Chung S.H., Kim Y., Kim B.S., Kim H., Beuchat L.R., Ryu J.H. (2015). Behaviour of *Aspergillus flavus* and *Fusarium graminearum* on rice as affected by degree of milling, temperature, and relative humidity during storage. Food Microbiol..

